# Family members, ambulance clinicians and attempting CPR in the community: the ethical and legal imperative to reach collaborative consensus at speed

**DOI:** 10.1136/medethics-2020-106490

**Published:** 2020-10-15

**Authors:** Robert Cole, Mike Stone, Alexander Ruck Keene, Zoe Fritz

**Affiliations:** 1 West Midlands Ambulance Service NHS Foundation Trust, Brierley Hill, UK; 2 Coventry, UK; 3 Dickson Poon School of Law, Kings College London, London, UK; 4 THIS institute (The Healthcare Improvement Studies Institute), University of Cambridge School of Clinical Medicine, Cambridge, UK; 5 Acute Medicine, Cambridge University Hospitals NHS Foundation Trust, Cambridge, UK

**Keywords:** right to refuse treatment, autonomy, clinical ethics, education for health care professionals, end-of-life

## Abstract

Here we present the personal perspectives of two authors on the important and unfortunately frequent scenario of ambulance clinicians facing a deceased individual and family members who do not wish them to attempt cardiopulmonary resuscitation. We examine the professional guidance and the protection provided to clinicians, which is not matched by guidance to protect family members. We look at the legal framework in which these scenarios are taking place, and the ethical issues which are presented. We consider the interaction between ethics, clinical practice and the law, and offer suggested changes to policy and guidance which we believe will protect ambulance clinicians, relatives and the patient.

## Introduction

Internationally, pre-hospital registered ambulance clinicians (variously called ambulance clinicians, paramedics and emergency services personnel) are often put in the invidious position of having to make a decision about whether or not to attempt cardiopulmonary resuscitation (CPR) when they attend a call and find a patient whose heart has stopped. About 46% of deaths in the England occur in homes or nursing homes^1^ and ambulances are often called at times of health crisis, even when a death is expected, if caregivers feel unsure what to do.^2^ The call has been put out, the ambulance clinician has responded to the call: to do nothing creates certainty around the individual’s death. Where the heart stopping is the final stage of a longer dying process, attempting CPR is likely to be futile, as the heart stopping reflects an overall physiological deterioration which CPR cannot reverse. In other circumstances, particularly in cases where the arrest is unexpected and the primary problem is with the heart, it may result in full recovery for the individual. Or it may give the individual a chance of returned circulation, but with great neurological deficit;^3^ or it may restart the heart briefly, only for the individual to die again.^4^

The ambulance clinician must therefore make a rapid decision with potentially very significant repercussions. To protect them from the emotional work—and possible litigation—associated with these decisions, their recently updated UK professional guidance^5^ recommends: “Where no explicit decision about CPR has been considered and recorded in advance, there should be an initial presumption in favour of CPR.” Clinicians are, however, given the discretion to make decisions not to attempt CPR where they think it would be futile, ‘for example, for a person in the advanced stages of a terminal illness where death is imminent and unavoidable’. However, there is no explicit mention of the importance of listening to family members’ views of what the patient would want, nor reference to the legal obligation of the ambulance clinician to follow the Mental Capacity Act 2005 (MCA 2005) and do what is in the patient’s best interests (which would involve taking into consideration what family members/friends and advocates think the patient would want). In the USA, guidance is not included on how to incorporate relatives’ views with best interests decisions. Ambulance clinicians have reported that they have not been taught to deal with these decisions^6^ and that it is often easier for them—both emotionally and logistically—to deliver attempted CPR than to consider withholding it. Relatives, who, after all, have been the ones to place the call in the first place, then feel powerless (and sometimes angry) when ambulance clinicians start CPR despite their protestations that this is ‘not what he/she would have wanted’. In the USA, emergency services personnel have even less discretion than in the UK: in many states, they are bound to start CPR unless a specific Do Not Attempt Cardiopulmonary Resuscitation (DNACPR) is in place, even if the patient has another kind of documentation, for example POLST (Physician Order for Life-Sustaining Treatment) until they have spoken to a ‘medical command physician’. They also must continue CPR if it has been started by a bystander even if a DNACPR is in place, until they are told they can stop by a physician.

To highlight the moral discomfort experienced and the ethical and legal challenges faced, we present the perspectives of an ambulance clinician and a relative, and then review the legal and ethical framework in which they are operating, before concluding with some suggested changes to policy and guidance which we believe will protect ambulance clinicians, relatives and the patient.

## Ambulance clinician’s perspective—Rob Cole

The following is a case study to illustrate the grey area faced by ambulance clinicians when they consider they need to make a ‘best interests’ decision on a patient who has arrested. This is a composite case study from my experience of many such calls to protect the anonymity of those involved in any individual case.

An emergency call was received by the ambulance emergency operations control room. At this stage, it was important to clarify the justification for this call as this directly influences any further decision making. If the call was for the purpose of providing resuscitation to a patient in cardiorespiratory arrest then, as early as this stage, we can determine that at the point of call, somebody (accepting unable to qualify exactly whom) believes that the patient is either clinically indicated for resuscitation or someone believes they would desire or benefit from such an intervention. The caller identified that her husband was experiencing a seizure, and this had lasted for 5 min prior to her calling the ambulance. An ambulance was immediately despatched on this information alone (known as pre-alert dispatch). The location was some 4 min from the crew and they therefore arrived on the scene 5 min post call (in fact, on the crew arrival, the caller was still on the phone with the ambulance control centre).

The crew were met by a female in her 70s (call with control ended on crew arrival). The crew were, as often is the case, provided with no further details other than that of a male in his 80s with a prolonged seizure. The ambulance had travelled under emergency conditions to the address. The female greeted the crew (who had approached the property with full life-saving emergency equipment). She stated “I think he has gone” in a calm and clear voice. She allowed the crew into her home and quickly explained (during the journey to the patient, who is on a bed in the dining room downstairs) that the patient was her husband, that he had been generally unwell for some time (increased frailty, heart failure and developing dementia) and while she had not expected him to die at this point in time, she was not particularly surprised that he had. One member of the crew (double crew) prepared the patient for resuscitation, post a period of assessment while the other crew member continued to speak with the patient’s wife to better understand the situation. The scene looked non-suspicious: the patient was lying peacefully (not breathing and with no heart rate) on a bed downstairs, dressed in pyjamas. The patient presented as frail in appearance but other than that, there was no further information of note.

The member of the crew that spoke with the wife of the patient and ascertained that the patient was being treated by a general physician for a simple urinary tract infection, that there was no DNACPR in place as there was no specific requirement for one to have been put in place; no advance decision to refuse treatment (the female had no idea what this was) nor was there any legal power of attorney (the patient until this point had been broadly of sound mind with occasional episodes of confusion). As the other member of the ambulance crew commenced resuscitation (CPR), the patient’s wife angrily stated that her husband would not wish for this, nor did she or any member of her family. She reiterated that the 999 call was due to a seizure, and had it been for the purpose of providing resuscitation, she would not have called the emergency services and all agreed that this was not the wish of the patient. Accepting this is not documented anywhere, the patient’s wife explained that these were conversations that had taken place within the family environment, that her husband had a clear view that he would not want to be subjected to any resuscitative efforts should he die, and funeral arrangements had been explored recently by all.

To add, the patient’s wife appeared to be of sound mind, no obvious level of confusion and not in any particular state of heightened distress. The son of the patient was 10 min away from the address and on his way. A neighbour had also arrived at the property.

To summarise, cardiac arrest of a patient in his 80s, not expected to die but family not surprised (had been quite unwell recently), no DNACPR or other documented evidence of the patient’s thoughts, wishes and beliefs. Call for emergency help was to manage a seizure and NOT provide resuscitation.

## Family carer perspective—Mike Stone

When my mother died about 10 years ago,^7^ I might have found myself as a relative trying to prevent a 999 paramedic from attempting CPR, but in the event, I found myself being ‘confronted by’ 999 personnel who seemed unable to understand why when my mum died at the end of a peaceful 4-day terminal coma, I had NOT felt the need ‘to phone someone immediately’. This prompted me to embark on an investigation into end-of-life (EoL) guidance, protocols, mindsets and laws, which revealed to me a situation I can, at best, describe as urgently requiring improvement, especially but not exclusively for EoL-at-home, and which, in complex and confusing situations, protects professionals at the expense of damaging relatives and, sometimes, even patients.

From my family carer perspective, this situation has to change. And, the direction of change must be one which improves the support given to patients, by promoting integration between everyone, lay and professional, involved in supporting patients. This ‘model’ requires ‘us and us’ as opposed to ‘us and them’: it emphasises teamwork between family carers and the clinicians who are in regular and ongoing contact with the patient, and it replaces ‘multidisciplinary team thinking’, with genuine professional-lay integration.

Anyone can listen to a patient—provided you are present to listen: if only a relative is present, only the relative can listen. Often it will require a clinician, such as a 999 paramedic, to confirm that a patient is in cardiopulmonary arrest, but the family carer who called 999, is the person most likely to know if the patient would have wanted CPR. Put simply, the clinicians are the experts in the clinical aspects, and the family and friends are the experts in ‘the patient as an individual’.

I believe the current guidance around CPR decision-making is unsatisfactory and incoherent, and must be made more sensible and coherent.^8–10^ Contemporary protocols for ‘expected death’ are also fundamentally flawed.^11^ Advance decisions often fail to achieve the patient’s objective, apparently because clinicians are risk-averse.^12^

I have only mentioned a few of the more significant problems, and those I have mentioned could, in theory, be addressed by consensus followed by improved training. Other fundamental problems—notably the fact that relatively few people have personal experience of caring for a loved one all the way to a death at home—are more problematic.

To close this brief and personal analysis, I will give two opinions. The first is that the change required is easy to see, and involves things such as more group-based and ‘diffusely achieved’ decision-making instead of identifiable individuals being invariably associated with and responsible for specific decisions. But it is a change which a hierarchical and process/records-based National Health Service (NHS) would really struggle to come to terms with.^13^

The second is my optimism that growing pressure from patients and relatives will make the changes in behaviour inevitable, because, perhaps surprisingly, of social media.^14^

## Legal analysis—Alex Ruck Keene

Mike’s experiences speak clearly of the practical problems caused by paramedics misunderstanding the law.

If there is a situation in which CPR would simply not work to restart the heart or breathing, then the paramedics would be under no duty to attempt it, as there is no duty to seek to carry out a futile procedure. However, if it appeared that it might work, then the paramedics are, in England and Wales, governed by the MCA 2005. In practice, the realities confronted by paramedics are such that the majority of their decision-making will be governed by the MCA 2005. This Act provides a framework for decision-making in relation to those with impaired decision-making capacity which is (unlike legal frameworks in some other jurisdictions) not predicated on there being an automatic proxy decision-maker, such as a ‘next of kin.’ Rather, the Act provides (in s.5) that any person—such as a paramedic—is able to carry out an act of care and treatment in relation to another (‘P’) with protection from liability if they: (1) take reasonable steps to determine whether P has the capacity to consent to the act; and (2) if P lacks capacity, that they reasonably believe that they are acting in P’s best interests.

In all situations, the first step is to consider whether the person has capacity to make their own decision—to consent to or refuse CPR. In the scenario presented by Rob Cole, as with almost all situations where CPR is required, the patient was unconscious and there were no practicable steps that could be taken to support him within the time available: reaching the conclusion that the patient did not have capacity could therefore have been effectively instantaneous.

The paramedics had taken reasonable steps to ascertain whether the person had made an advance decision to refuse CPR (as a medical treatment), and that he had not made one.

This means that they were therefore required to decide whether it was in his best interests for them to attempt it.

‘Best interests’ is, deliberately, not defined in the MCA 2005. However, s.4 sets out a series of matters that must be considered whenever a person is determining what is in the person’s best interests to allow them to have a reasonable belief as to they are acting in those best interests. It is extremely important to recognise that the MCA 2005 does not specify what is in the person’s best interests. Rather, it sets down a process by which that conclusion should be reached, which recognises that a lack of decision-making capacity is not an ‘off-switch’ for their rights and freedom (Wye Valley NHS Trust v- Mr B ]2015[ EWCOP 60 in paragraph 11). The process aims to construct a decision on behalf of the person who cannot make that decision themselves. As the Supreme Court emphasised in Aintree University NHS Hospitals Trust v James [2014] UKSC 67 “[t]he purpose of the best interests test is to consider matters from the patient’s point of view.” It is critically important to understand that the purpose of the decision-making process is to try to arrive at the decision that is the right decision for the person themselves, as an individual human being, and not the decision that best fits with the outcome that the professionals desire. Any information about the patient’s wishes, feelings, beliefs and values will be relevant, including, in particular, preferences and recommendations documented when the person had capacity.

Consultation will also be required with those who could shed light on the person’s likely decision, here his wife. The case of Winspear v City Hospitals Sunderland NHS Foundation Trust [2015] EWHC 3250 (QB) made clear that a failure to consult where it is practicable and appropriate will mean that professionals cannot then rely on the defence in s.5 of MCA to what might otherwise be criminal acts.

In making a best interests decision about giving life-sustaining treatment, there is always a strong presumption that it will be in the patient’s best interests to prolong his or her life, and the decision-maker must not be motivated by a desire to bring about the person’s death for whatever reason, even if this is from a sense of compassion. However, the strong presumption in favour of prolonging life can be displaced where:

- There is clear evidence that the person would not want the treatment in question in the circumstances that have arisen.
- The treatment itself would be overly burdensome for the patient, in particular by reference to whether the patient accepts invasive and uncomfortable interventions or prefers to be kept comfortable.
- There is no prospect that the treatment will return the patient to a state of a quality of life that the patient would regard as worthwhile. The important viewpoint is that of the patient, not of the doctors or healthcare professionals.

Case law has made clear that the weight that is to be attached to the reliably ascertainable views of the person should be given very substantial, if not determinative, weight (Re AB (Termination of Pregnancy) [2019) EWCA Civ 1215]. In a case such as that described in the scenario of the ambulance clinician, and given the clarity of the views expressed by the man’s wife in relation to what he would have wanted, the paramedics could properly conclude that attempting CPR was not in his best interests. The Supreme Court has confirmed that they should not then attempt it: NHS Trust v Y [2018] UKSC 22.

Drawing the legal threads together, therefore, in a situation such as this:

1. Unless the paramedics have a proper reason to doubt the good faith of the family member present, they should proceed on the basis that they are reliable in relaying what the person would have wanted.
2. The paramedics can then either start or not start CPR accordingly because they have the necessary reasonable belief that they are acting in the person’s best interests.
3. If there is reason to doubt the good faith of the family member present, or the family member does not (or cannot) relay clear views, the paramedics should start CPR. It may be that after they have started, they are able to glean further information which makes the picture clearer and enables them to decide whether continuing is in the patient’s best interests.

## Ethical overview and proposals for change—Zoë Fritz (and other authors)

Law, ethical principles and professional clinical guidelines influence each other.^15^ In an ideal system, this would ensure just care with recognition of the rights of practitioners and patients. When it works badly, the ‘letter of the law’ is followed, even when it runs counter to good ethics, with potentially devastating personal consequences. The composite scenario and personal events, described above by an ambulance clinician and a family member, reflect examples of where medical practitioners believed they were following the law, but where their actions could be argued to have been unethical.

In contrast, a related example of the law working positively to overturn accepted clinical guidance and practice, is around the need to discuss a decision not to attempt CPR with a patient. The 2007 joint guidance issued by the British Medical Association, Royal College of Nursing and the Resuscitation Council (UK) (2007) stated: “When a clinical decision is made that CPR should not be attempted, because it will not be successful, and the patient has not expressed a wish to discuss CPR, it is not necessary or appropriate to initiate discussion with the patient to explore their wishes regarding CPR.” The case of Janet Tracey challenged this: the judges in the court of appeal found that not discussing a decision to withhold CPR with a patient was in breach of their human rights (Article 8 European Convention on Human Rights) as it deprived them of the right to question the clinical decision or ask for a second opinion, particularly in the context of a potentially life-saving treatment.^16^ Clinicians rapidly changed their practice; in fact, the whole nature of CPR conversations was altered to ensure that it was not considered in isolation, but always discussed within overall goals of care; in being forced to discuss CPR with patients, doctors reconsidered the conversation, what it meant and when it could and should occur.^17^

The ReSPECT (Recommended Summary Plan for Emergency Care and Treatment) process emerged from this as a way of nudging doctors and patients into having better conversations and documentation of agreed recommendations;^18^ it is now used in more than 130 trusts.^19^

While, at first glance, there may appear to be ethical and legal tensions in the scenarios described above, it is possible that good training and professional guidance would dispel them. If families were better supported to understand what may happen where a loved one dies at home, they would be better equipped to deal with the crisis when it came; specific resources are needed. If, for example, there had been a specific number to call for an expected death, other than 999, in the two deaths reported here, then neither of these upsetting scenarios would have occurred. As mentioned above, social media may be another positive force in both applying pressure for change, and in acting as a leveller in terms of access to information.

If the professional guidance and other material—published by Joint Royal Colleges Ambulance Liaison Committee, Royal College of Nursing, Resuscitation Council UK and so on—stated clearly that, where death was expected and CPR appeared to be futile, even in the absence of a DNACPR or ReSPECT form, an ambulance clinician or qualified nurse could decide that attempting CPR was clinically pointless or potentially harmful, then clinicians would not need to choose between what they considered morally right and what they had to do to protect their professional registration.

The new JRCALC guidance takes this into account, and it is likely that other guidance will also be explicit about this in the future; they should also be explicit about the role of the MCA and best interests decisions. An honest carer, family member who protests, “… but my husband would definitely not want CPR—don’t do that!” may be perceived as applying the MCA to her own determination of what is in her husband’s best interests, even if the wife has no awareness of the MCA.

If the ambulance clinicians were taught clearly that acting in the patient’s ‘best interests’ in this scenario most often meant doing as the relatives asked, then the (frequently internalised) concern that they were choosing between what was right for the patient and what was right for the patient’s relative would be abolished, and the associated moral discomfort diminished. We recognise that there will, in some cases, be a different tension—where the ambulance clinician considers that the CPR will not be successful but the relatives want it to take place. But this is where the distinction between the ambulance clinician as the expert in the medical procedure and the relative as the expert in the person comes in—nobody can demand medical treatment which is inappropriate, and CPR is no different. The guidance and the training should emphasise the teawork which Mike Stone mentions above: the default assumption should be that clinicians and relatives have a shared goal of what is best for the patient, and work together as ‘us and us’ as opposed to ‘us and them’.

## Data Availability

There are no data in this work.

## References

[1] Public Health England. Palliative and end of life profiles, 2017.

[2] Ingleton C, Payne S, Sargeant A, et al. Barriers to achieving care at home at the end of life: transferring patients between care settings using patient transport services. Palliat Med 2009;23(8):723–30. 10.1177/0269216309106893 19643950

[3] Ebell MH, Jang W, Shen Y, et al. Development and validation of the good outcome following attempted resuscitation (GO-FAR) score to predict neurologically intact survival after in-hospital cardiopulmonary resuscitation. JAMA Intern Med 2013;173(20):1872–8. 10.1001/jamainternmed.2013.10037 24018585

[4] National Confidential Enquiry into Patient Outcome and Death. Time to Intervene? A review of patients who underwent cardiopulmonary resuscitation as a result of an in-hospital cardiorespiratory arrest [Online], 2012. Available: http://www.ncepod.org.uk/2012cap.htm

[5] Joint Royal Colleges Ambulance Liaison Committee. Clinical guidelines, 2019.

[6] Moffat S, Fritz Z, Slowther A-M, et al. ‘Do not attempt CPR’ in the community: the experience of ambulance clinicians. J Paramedic Pract 2019;11(5):198–204. 10.12968/jpar.2019.11.5.198

[7] Stone M. What I learnt from the deaths of my parents – 'how I became involved' in EoL debate, 2018. Available: https://www.dignityincare.org.uk/Discuss-and-debate/download/315/

[8] Stone M. Rapid response to David Oliver’s article’ Resuscitation Orders and reality. Available: https://www.bmj.com/content/352/bmj.i1494/rr-3

[9] Stone M. I have a suggestion for how family-carers and 999 paramedics 'could be reconciled' for CPR decision-making - feedback from family-carers welcomed, 2019. Available: https://www.dignityincare.org.uk/Discuss-and-debate/Dignity-Champions-forum/I-have-a-suggestion-for-how-family-carers-and-999-paramedics-could-be-reconciled-for-CPR-decision-making-feedback-from-family-carers-welcomed./1031/

[10] Stone M. Mike’s Cheeky Blog: I believe that CPR should be attempted if a mentally-capable patient had asked for CPR to be attempted, 2019. Available: https://www.dignityincare.org.uk/Discuss-and-debate/Dignity-Champions-forum/Mikes-Cheeky-Blog-I-believe-that-CPR-should-be-attempted-if-a-mentally-capable-patient-had-asked-for-CPR-to-be-attempted/1051/

[11] Stone M. An end-of-life Timeline with an emphasis on death at home. Available: https://www.dignityincare.org.uk/Discuss-and-debate/download/275/

[12] Stone M. Rapid response to “ we need to talk about resuscitation”, 2017. Available: https://www.bmj.com/content/356/bmj.j1216/rr-1

[13] Stone M. Rapid response to: Are you ready for “collaborative health”? 2017. Available: https://www.bmj.com/content/358/bmj.j3257/rr-4

[14] Stone M. BMJ supportive and palliative care, 2018. Available: https://blogs.bmj.com/spcare/2018/09/14/how-social-media-expanded-my-world-by-a-bereaved-carer/

[15] Foster C, Miola J. Who's in charge? the relationship between medical law, medical ethics, and medical morality? Med Law Rev 2015;23(4):505–30. 10.1093/medlaw/fwv004 25752596

[16] R (Tracey) v Cambridge University Hospital NHS Foundation Trust and others [2014] EWCA Civ 822.

[17] Fritz Z, Cork N, Dodd A, et al. DNACPR decisions: challenging and changing practice in the wake of the Tracey judgment. Clin Med 2014;14(6):571–6. 10.7861/clinmedicine.14-6-571 PMC4954125 25468838

[18] Fritz Z, Slowther A-M, Perkins GD. Resuscitation policy should focus on the patient, not the decision. BMJ 2017;356. 10.1136/bmj.j813 PMC5330195 28246084

[19] ReSPECT (Recommended Summary Plan for Emergency Care and Treatment). Available: https://www.resus.org.uk/respect

